# Supplementary material for: Activation and inactivation pathways of a p53-like transcription factor govern lipid homeostasis in yeast
Source: bioRxiv. 2026 Sep 2:2026.08.31.748402. Preprint. [Version 1] doi: 10.64898/2026.08.31.748402 (PMC13684490; doi:10.64898/2026.08.31.748402)
Supplement: Supplementary file 1 [file media-1.pdf]

Supplementary Materials for

Activation and inactivation pathways of a p53-like transcription factor govern lipid homeostasis in yeast

Neng Wan *et al.*

*Corresponding author. Email: tom_rapoport@hms.harvard.edu

This PDF file includes:

Figs. S1 to S4
Tables S1 to S3

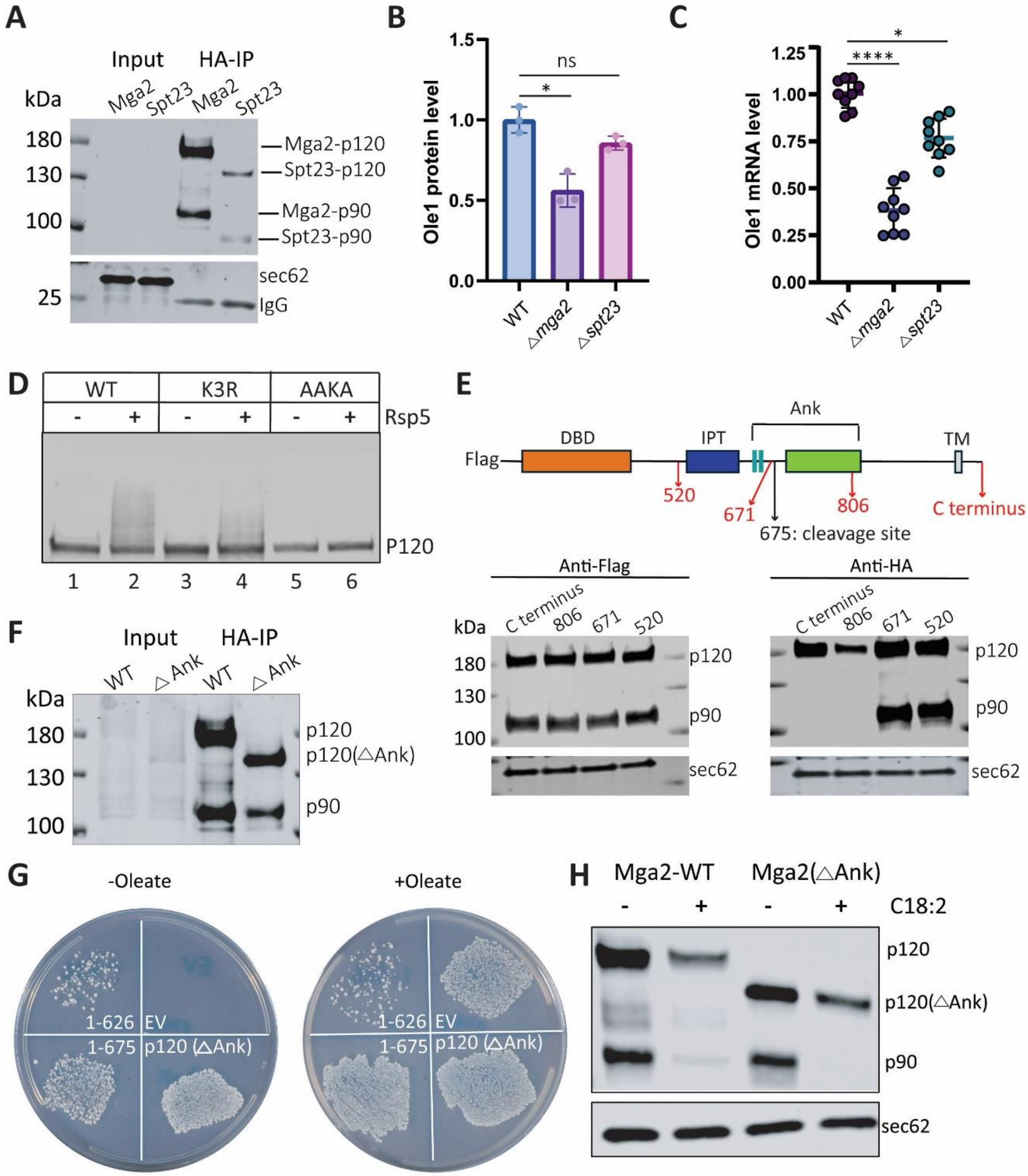

Figure S1. Processing of the Mga2 precursor.

(**A**) Mga2 or Spt23 with N-terminal 2xHA tags were expressed from the chromosome. Triton-solubilized cell lysates were incubated with HA-antibody beads and the bound material was analyzed by SDS-PAGE and immunoblotting with HA antibodies. Sec62 and immunoglobulin (IgG) served as loading controls. (**B**) RNA was extracted from wild-type (WT) cells or cells lacking Mga2 or Spt23 (∆mga2 or ∆spt23) and the level of Ole1 mRNA determined by RT-

qPCR. The levels of Ole1 transcripts are expressed relative to those of WT. (**C**) Ole1 was tagged at the C-terminus with 2xHA epitopes and expressed in WT, ∆mga2, or ∆spt23 cells. Cell lysates were analyzed by SDS-PAGE and immunoblotting with HA antibodies. The intensity of the Ole1-2xHA bands was quantified and is expressed relative to that of WT. (**D**) Full-length wild-type (WT) Mga2 or mutants with three lysine residues mutated (K3R: K980R, K983R, K985R) or defective in Rsp5 binding (AAKA) were Flag-tagged at the N-terminus and over-expressed in ∆mga2 cells. Cell lysates were subjected to immunoprecipitation with anti-Flag beads. After washing, the beads were incubated with purified Rsp5 and the bead-bound material analyzed by SDS-PAGE and immunoblotting with Flag antibodies. Controls were performed in the absence of Rsp5. (**E**) The HA epitope was inserted at the indicated positions into Flag-tagged full-length Mga2, and the proteins were over-expressed in ∆mga2 cells. Cell lysates were analyzed by immunoblotting with Flag and HA antibodies. Blotting for Sec62 served as a loading control. (**F**) Full-length Mga2 carrying N-terminal 2xHA tags was expressed from the chromosome. Cell lysates were subjected to immunoprecipitation with HA-antibody beads. The samples were analyzed by SDS-PAGE and immunoblotting with HA antibodies. (**G**) Mga2 fragments containing residues 1-675 or 1-626 with an N-terminal Flag-tag were over-expressed in cells lacking both Mga2 and Spt23. Where indicated, cells expressed from a centromeric plasmid a p120 variant that lacks the Ank domain. An empty vector (EV) was used as control. The cells were grown on plates lacking or containing oleate at 30°C for three days. (**H**) As in (F) but with addition of unsaturated fatty acid C18:2, as indicated.

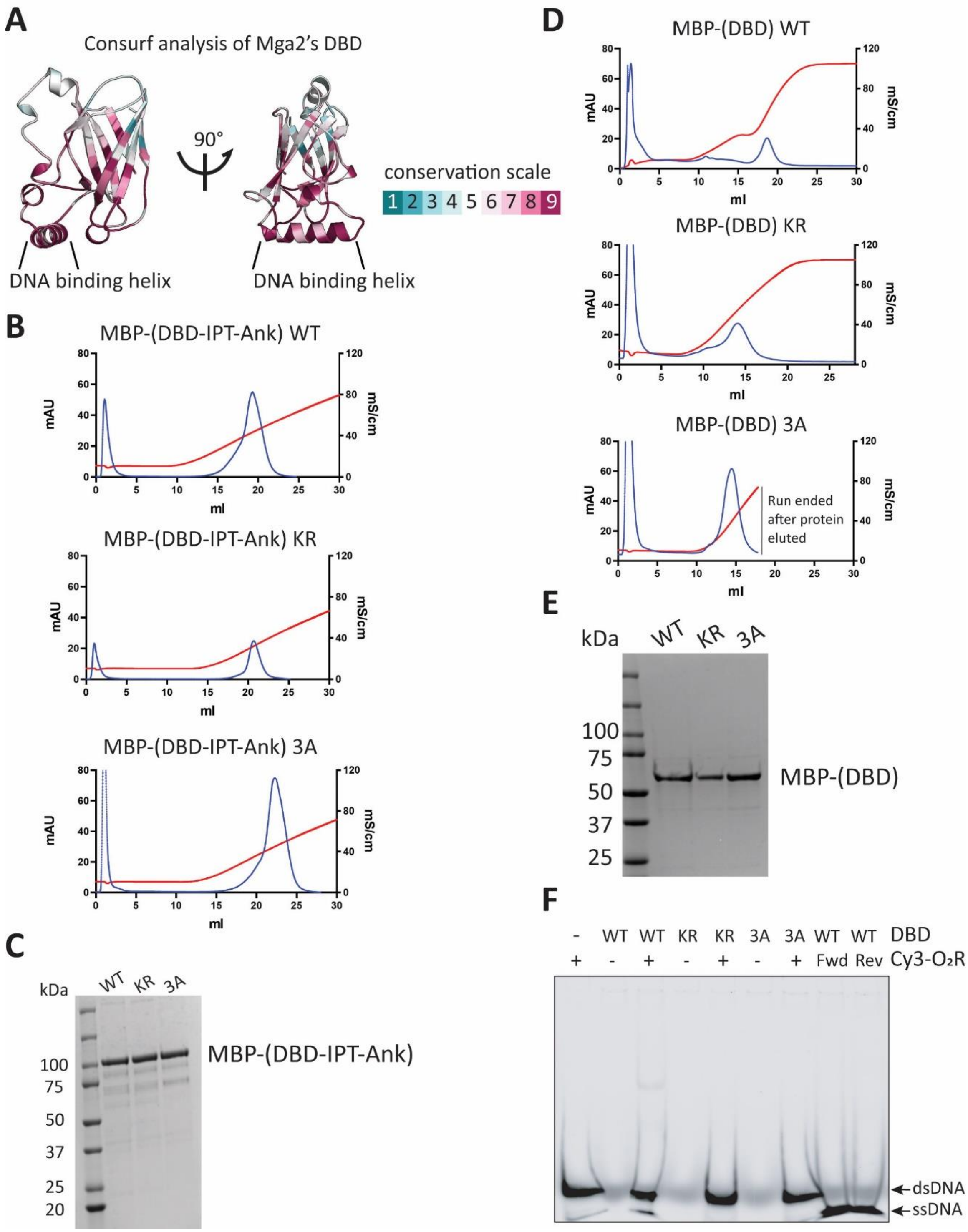

Figure S2. Structural conservation of Mga2's DBD and purification of DBD-containing proteins.

(**A**) Conservation of the DBD as analyzed by the program Consurf. The AlphaFold-predicted structure was used as input. The scale of conservation is shown on the right. Note that the

DNA binding helix is highly conserved. (**B**) Mga2 fragments spanning the region from the DBD to the Ank domain were expressed as fusions with the maltose-binding protein (MBP) in E. coli (MBP-(DBD-IPT-Ank). The DBD domain was either wild-type (WT) or contained the KR or 3A mutations. The proteins were purified on an amylose resin and applied to a heparin column. Bound protein was eluted with a linear salt gradient. (**C**) The purified proteins in b were subjected to SDS-PAGE and Coomassie-blue staining. (**D**) As in (B), but with MBP-fusions of the DBD alone. (**E**) As in (C), but with MBP-DBD fusions. (**F**) Gel-shift assays using MBP-DBD fusions and Cy3-labeled O2R DNA. The experiment was performed as in Fig. 2D.

Figure S3

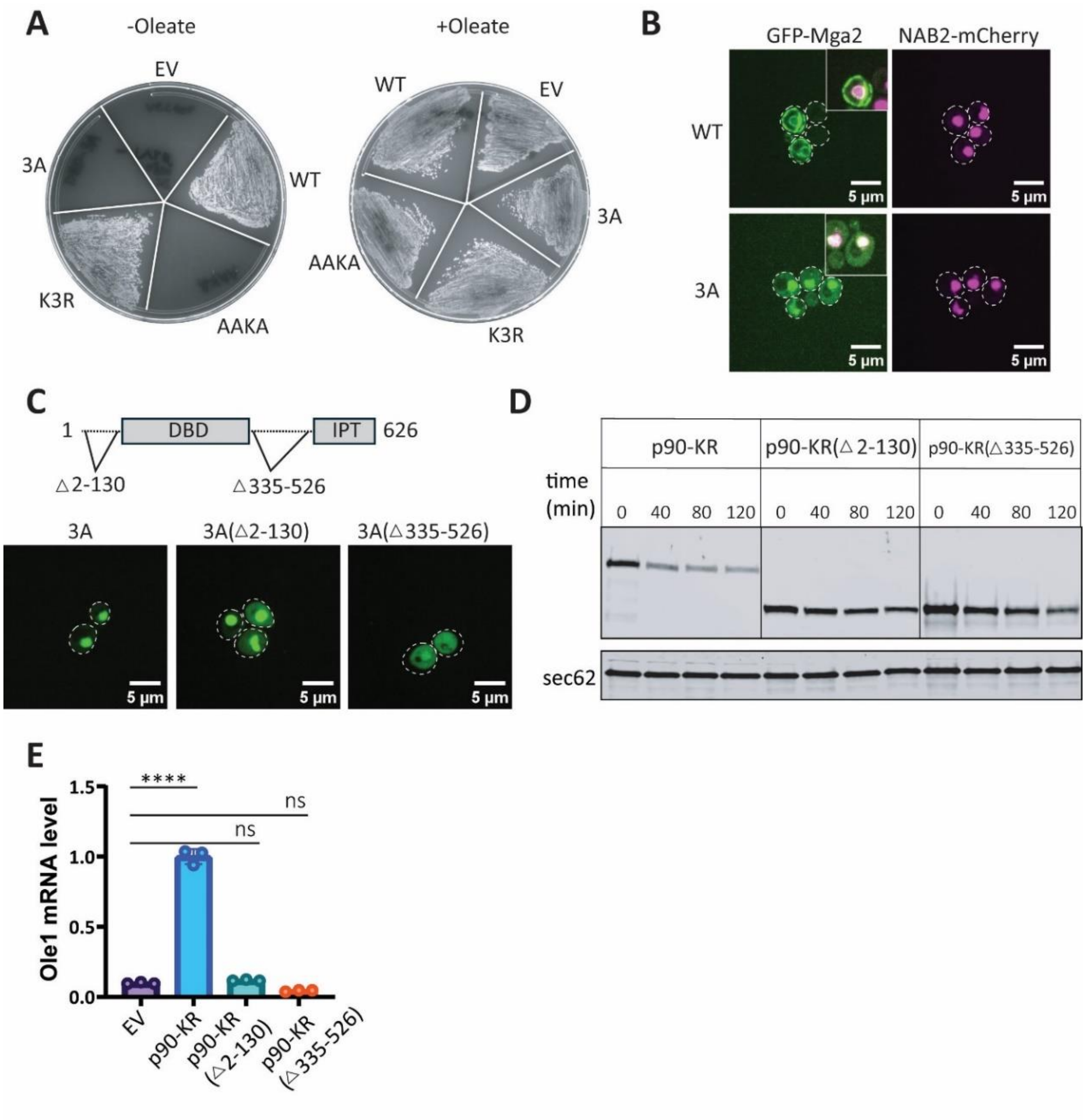

Figure S3. Degradation of p90 requires nuclear import and DNA binding.
(**A**) Full-length wild-type (WT) Mga2 or the indicated mutants were expressed from the native promoter on a centromeric plasmid in cells lacking Mga2 and Spt23. The cells were grown on plates lacking or containing oleate at 30°C for three days. Controls cells received an empty vector (EV). (**B**) GFP-tagged full-length Mga2 or the corresponding 3A mutant were expressed from the native promoter on a 2m plasmid. The cells also stably expressed NAB2-mCherry as a nuclear marker. GFP and mCherry fluorescence was analyzed by confocal microscopy. The inset shows merged images. The boundaries of the cells are indicated. (**C**) GFP-tagged p90 (residues 1-626) containing the 3A mutation (3A) or variants that lack residues 2-130 or 335-526 (3A (Δ2-130) and 3A (Δ335-526), respectively) were expressed from the native promoter on a 2μ plasmid in Δmga2 cells. GFP fluorescence was analyzed by confocal microscopy. The boundaries of the cells are indicated. (**D**) HA-tagged p90 carrying the KR mutation (p90-KR; residue 1-626) or variants lacking residues 2-130 or 335-526 (p90-KR (Δ2-130) and p90-KR

(∆335-526) were over-expressed in ∆mga2 cells. Cycloheximide was added, and samples taken at different time points were analyzed by SDS-PAGE and immunoblotting with HA antibodies. (**E**) RNA was extracted from the indicated cells and Ole1 mRNA levels were quantified by RT-qPCR. The levels of Ole1 transcripts are expressed relative to those of p90-KR.

Figure S4

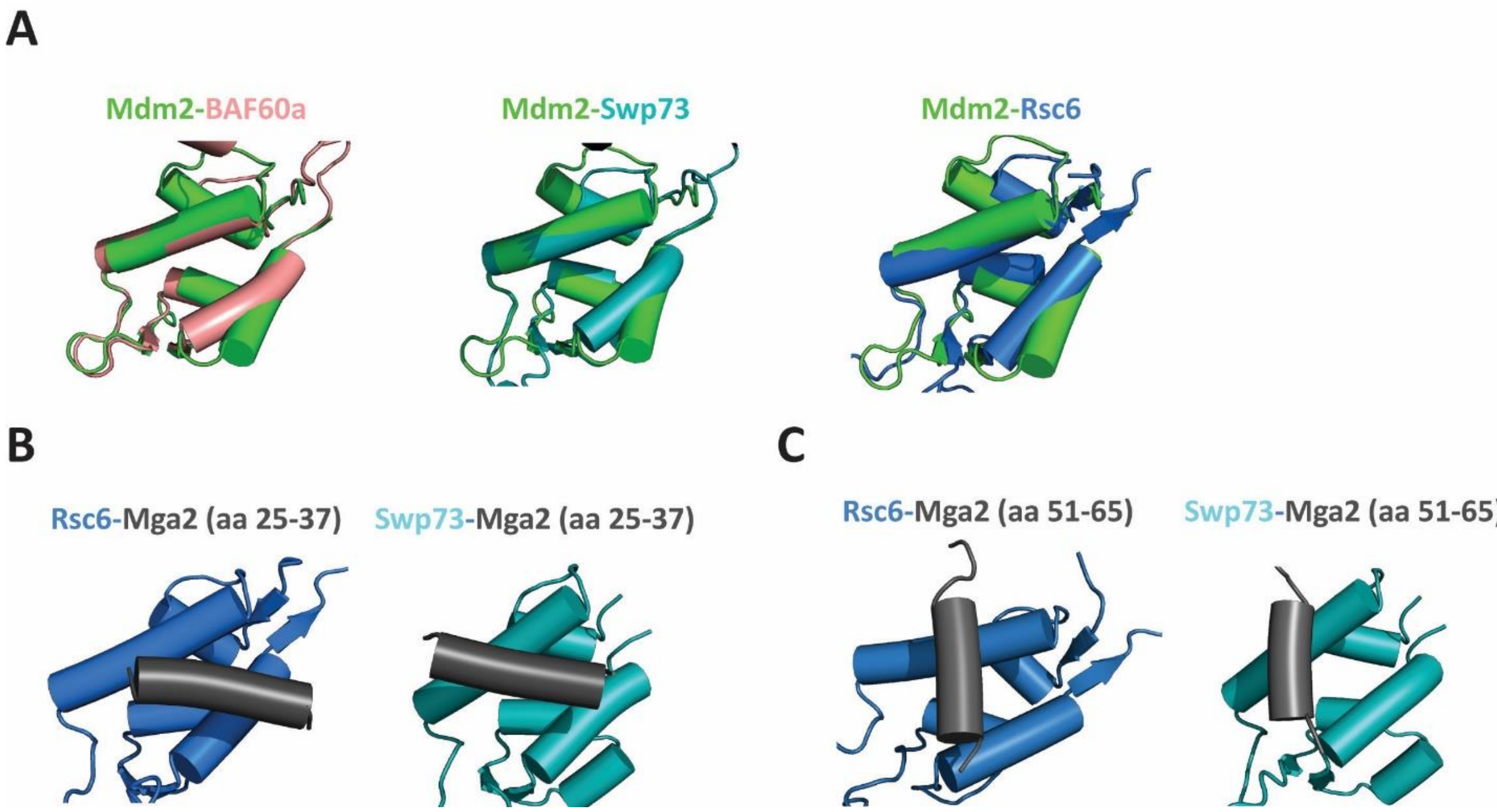

Figure S4. Predicted interactions of Mga2 with chromatin remodeling components.
(**A**) Structural comparison of the TAD1-binding domain of Mdm2 with domains of chromatin remodeling complexes. BAF60a (also called SMARCD1) and Swp73 are components of the human and yeast SWI/SNF complexes, respectively (r.m.s.d: 0.91Å and 0.77Å), and Rsc6 is a component of the yeast RSC complex (r.m.s.d: 2.11Å). (**B**) AlphaFold predictions of complexes of the TAD-like sequence of Mga2 (dark grey, residues 25-37) and the binding domains of Rsc6 and Swp73 (iPTM=0.53 and 0.62, respectively). (**C**) AlphaFold predictions of complexes of residues 51-65 of Mga2 (dark grey) and the binding domains of Rsc6 and Swp73 (iPTM=0.64 and 0.59, respectively).

Table S1: primers used in the study

Name	Sequence (5'-3')
GU_Mga2_F	AGGAGAAAAAACCCGGGTACCATGGACTACAAAGACCATG
GU_Mga2_R	CCGCTTATTTAGAAGTGTCGAATTCCTAACTGACAATTAAATCGTTC
GL_RSP5_F	AGTAAGAATTTTTGAAAGGGGATCCATGTACCCATACGATGTTCCAGATTACGCTCCTTCATCCATATCCGTC
GL_RSP5_R	AAAGCTTGCATGCCTGCAGGTCGACTCATTCTTGACCAAACCCTATGGTTTCTTCC
pET28-DBD_F	CCTGTATTTCCAGGGAGAAGGATCCTACATAAATCCTTTAGATTACCTTAAAG
pET28-DBD_R	TGGTGGTGGTGCTCGAGTGCGGCCGCTCATGTCGTAAACTTACCCAACAG
K254R_F	CGCTAGAAGGCGCTCAGGTATT
K254R_R	GCTCTTCTTTGTTCTCGC
3A_F	TGCAGCTGCGTCAGGTATTGCAGACAATTTACTCTGGT
3A_R	GCGGCTCTTCTTTGTTCTCGCTTAAC
pET28_MBP_F	ATTTCCAGGGAGAAGGATCCGACTACAAAGACCATGACGG
MBP_R	CCGTCATGGTCTTTGTAGTCGGATCCTTCTCCCTGGAAAT
pET28a_Mga2_F	CACCAAATTTACAGACAGCGTATAAGCTCGAGCACCACCA
Mga2_R	CGCTGTCTGTAAATTTGGTGCCGTTCTGGAATTCTTCAAA
MBP-K254R_F	CGCGTCGCCGATCAGGTATC
MBP-K254R_R	CAGCACGACGCTGCTC
MBP-3A_F	CGGCTTCAGGTATCGCAGATAACTTGC
MBP-3A_R	CTGCCGCAGCACGACGCTG
Cas9_donor_2xHA-Mga2	TTATTGAAGGTCATTTTGGCGAACAGAACATTTCGTTATGTACCCTTACGATGTGCCCGATTACGCCTACCCTTACGATGT GCCCGATTACGCC CAGCAGAACAGTGAGTTCTTAACT GAA ACACCTGGAAGCGACCCTCATATATCTCAATTGCACGCGA
Cas9_donor_2xHA-Spt23	AACGACTAATCACAACAGTAGTACACCACTGAAAATGATGTACCCTTACGATGTGCCCGATTACGCCTACCCTTACGATGT GCCCGATTACGCC AGT GGC ACA GCA AACGTTTCCTCGATGCTCCACAGCTATAGCGCCAACATAC
Cas9_donor_2xHA-Mga2 (ΔAnk)	ATTGAGATCAATGACAATAAAAAGGCCATATTTACCTATGTTGATAAGTTTACCGATAGTGTAGAAACAGACAGTAATTAT TCTATTAGC
Cas9_donor_Ole1-2xHA	CTGTTATCAAGGAAAGTAAGAACTCTGCTATTAGAATGGCTAGTAAGAGAGGTGAAATCTACGAAACTGCTAAGTTCTTTT ATCCATACGACGTACCAGATTATGCTTACCCTTACGATGTGCCCGATTACGCCTAAGTATCACATTACAATAACAAAACTG CAACTACCATAAAAAAAAATTGAAAAATCAT
Mga2_check_F	CTGGCACTTTGTCTCAGGGTTAGTG
Mga2_check_R	TTTACATTTAAAGTATATACATTATCGTTACGCTGAC

Spt23_check_F	TTTCGATGGTGTACGTTGTATCAG
Spt23_check_R	AAAGAAGATAAAATTCAAAAATGCAAAATAATAAAAAATGAATC
OLE_ C-HA_F	GATCTTCTCCGCTCACTGGCCATTGA
OLE_ C-HA_R	AGCCGCCTTGCATGGTGCTTTGTCATT
Ole1P_750_F1	TGGAGCTCCACCGCGGTGGCGGCCGCAAGGATTAGCGGATATGTAGTTCC
Ole1P_750_R1	CTTTCGACATCTTTGTTGTAATGTTTTAGTGCTG
Ole1P_750_F2	TACAACAAAGATGTCGAAAGCTACATATAAGGAACG
Ole1P_750_R2	GTGGCGCGCCTTAGTTTTGCTGGCCGCATC
Ole1p_FAR_F1	TGGAGCTCCACCGCGGTGGCGGCCGCGGGCATGTCCCGGGGTTA
Ole1p_FAR_R1	CTTTCGACATGCTGGGATACCCGAAATAGCTC
Ole1p_FAR_F2	GTATCCCAGCATGTCGAAAGCTACATATAAGGAAC
Ole1p_O2R_F1	TGGAGCTCCACCGCGGTGGCGGCCGCTTCTTTCGGACGTTGAACACTC
Ole1p_O2R_R1	CTTTCGACATTAGAAGCACACCTGGTTGGG
Ole1p_O2R_F2	TGTGCTTCTAATGTCGAAAGCTACATATAAGGAAC
2xHA_Mga2_F	TATCCATACGACGTACCAGATTATGCTCAGCAGAACAGTGAGTTC
Mga2_426_R	CGAATTCCTGCAGCCCGGGGGATCCTTTACATTTAAAGTATATACATTATCGTTAC
F131_F	TTTGGAAGAAAGGAGACG
2xHA_Q2_R	AGCATAATCTGGTACGTCG
TAD-4E_F	TGAGGAGCTGAACGGGTCTCCCATG
TAD-4E_R	TCGTCTTCTTCCTGTGATTCCATTACGCTATTCG
Mga2-416_F	TGGAGCTCCACCGCGGTGGCGGCCGCATCTTTGCTTCAAAGATTTTTTG
Mga2-416_R	CGAATTCCTGCAGCCCGGGGGATCCTTTACATTTAAAGTATATACATTATCGTTAC
AAKA_F	AAAGCTGAGGATCTGTTCCCGTT
AAKA_R	TGCTGCGTCATCATTAATTCGATGTAAAACTCT
K985_F	CGATTGCGAACCACAAATCAAGACAGTATTGTG
K980_R	ATCATCTCGACCCCAAGACAACGG
Ank_F	AAGTTTACCGATAGTGTAG
Ank_R	AACATAGGTAAATATGGCC
Mga2-TEF1_F	TAAGTTTTCTAGAACTAGTGGATCCATGGATTATAAAGATGATGATGATAAAC
Mga2-TEF1_R	GACATAACTAATTACATGACTCGAGCTAACTGACAATTAAATCGTTCAAC
TEF1_626_R	GACATAACTAATTACATGACTCGAGCTAATCAACATAGGTAAATATGGCC
HA-F520-F	TGCCGGATTATGCGTTCTCAATGAAAAACAACAAC

HA-F520-R	CATCATACGGATAATCTTGCATTGAATGTAAAGC
HA-STOP-F	TGCCGGATTATGCGTAGTCTGCTTTTTTACGTATATATATATATATATG
HA-STOP-R	CATCATACGGATAACTGACAATTAAATCGTTCAAC
HA-N671-F	TGCCGGATTATGCGAACAGCTGTTCAAAAAGC
HA-N671-R	CATCATACGGATAGCCATTTGTACCGCTATC
HA-K806-F	TGCCGGATTATGCGAAACAAGATAACAGAGACAAC
HA-K806-R	CATCATACGGATATTTGTCTATTTCCTTTGAATGAATG
NES_F	CTATGTTGATGGTGGCGGAAGCGGAGG
NES_R	GACATAACTAATTACATGACTCGAGCTAAATATCCAATCCGGCAAGTTTCAGG
NES	GGTGGCGGAAGCGGAGGGGGTTCAGGCGGTGGGAGTGGAGGTGGCTCCTTGGCTTTGAAGTTGGCTGGTCTTGACATCG GTTCTGGTGGTTCTGGTCTTGCCCTGAAACTTGCCGGATTGGATATTTAG
N527_F	AATAATTTGCCATCAATTAATCG
I335_R	GATCATAATAGGTGTTGTCG
Mga2-1113_F	TAGTCTGCTTTTTTACGTATATATATATATATATG
Mga2-626_R	ATCAACATAGGTAAATATGGC
Mga2-675_R	TGTGCTTTTTGAACAGC
Mga2-836_R	CGTACCGTTTTGGAATTC
Mga2-GFP_F1	AGGGAACAAAAGCTGGAGCTCATCTTTGCTTCAAAGATTTTTTGC
Mga2_GFP_R1	CTTTACTCATAACGAAATGTTCTGTTCG
Mga2_GFP_F2	ACATTTCGTTATGAGTAAAGGAGAAGAAC
Mga2_GFP_R2	CGAATTCCTGCAGCCCGGGGGATCCCTAACTGACAATTAAATCGTTC
GFP-1113_F	TAGGGATCCCCCGGG
GFP-626_R	ATCAACATAGGTAAATATGGCCTTTTTATTGTCATTGATC
Cy3-O2R_F	Cy3-TCGGACGTTGAACACTCAACAAACCTTATCTAGTGCCCAACCAGGTGTGC
Cy3-O2R_R	Cy3-GCACACCTGGTTGGGCACTAGATAAGGTTTGTTGAGTGTTCAACGTCCGA
O2R-Native_F	TCGGACGTTGAACACTCAACAAACCTTATCTAGTGCCCAACCAGGTGTGC
O2R-Native_R	GCACACCTGGTTGGGCACTAGATAAGGTTTGTTGAGTGTTCAACGTCCGA
O2R-Scrambled_F	TAGCATGCAACCGTCTCTAGTGTCACAACAGATACCCAGCGTAACTCAGT
O2R-Scrambled_R	ACTGAGTTACGCTGGGTATCTGTTGTGACACTAGAGACGGTTGCATGCTA
FAR_F	CCGGGGTTAGCGGGCCCAACAAAGGCGCTTATCTGGTGGGCTTCCGTAGA
FAR_R	TCTACGGAAGCCCACCAGATAAGCGCCTTTGTTGGGCCCGCTAACCCCGG
Ole1-qPCR_F	TGCTCTCTCTGGTAAAGTGCC

Ole1-qPCR_R	CACCACCGACAGCGTAGTAG
5s_qPCR_F	GTTGCGGCCATATCTACCAGAAAG
5s_qPCR_R	CGTATGGTCACCCACTACACTACT
URA3-qPCR_F	CATTGCGAAGAGCGACAAAGA
URA3-qPCR_R	ACCGGGTGTCATAATCAACCA
O2R-qPCR_F	AGTCGGCAGCTTTCTTTC
O2R-qPCR_R	CAAGACTCGTAGAAGCACAC

Table S2: plasmids used in the study

Number	Plasmid
1	pBEVY-GL HA-Rsp5
2	pBEVY-GU 3xFlag-Mga2
3	pET28-HIS-MBP-Mga2 (141-330)
4	pET28-HIS-MBP-Mga2 (141-330); K254R
5	pET28-HIS-MBP-Mga2 (141-330); R252A, R253A, K254A
6	pET28-MBP-3xFlag-Mga2(127-842); Δ371-529
7	pET28-MBP-3xFlag-Mga2 (127-842); Δ371-529; K254R
8	pET28-MBP-3xFlag-Mga2(127-842); Δ371-529; R252A, R253A, K254A
9	pML104-2xHA Mga2 (ΔAnk) gRNA
10	pML104-2xHA Mga2 gRNA
11	pML104-2xHA Spt23 gRNA
12	pML104-Ole1 2xHA gRNA
13	pRS413-FAR (100bp)-URA3
14	pRS413-O2R (50bp)-URA3
15	pRS413-Ole1p (750bp)-URA3
16	pRS416-2xHA Mga2-1-626
17	pRS416-2xHA Mga2-1-626; Δ2-130
18	pRS416-2xHA Mga2-1-626; L31E, L32E, L35E, L36E
19	pRS416-Flag Mga2
20	pRS416-Flag Mga2; K980R, K983R, K985R
21	pRS416-Flag Mga2; L967A, P968A, Y870A
22	pRS416-Flag Mga2; R252A, R253A, K254A
23	pRS416-Flag Mga2; ΔAnk (626-836)
24	pRS416-TEF1-Flag Mga2-1-626
25	pRS416-TEF1-Flag Mga2-1-626; K254R
26	pRS416-TEF1-Flag Mga2-1-626; R252A, R253A, K254A
27	pRS416-TEF1-Mga2
28	pRS426-Flag-Mga2-(D519_HA_F520)
29	pRS426-Flag-Mga2-(S1113_HA_STOP)
30	pRS426-Flag-Mga2-HA (G670_HA_N671)
31	pRS426-Flag-Mga2-HA (K805_HA_K806)
32	pRS426-2xHA Mga2-1-626
33	pRS426-2xHA Mga2-1-626; Δ2-130
34	pRS426-2xHA Mga2-1-626; (LALKLAGLDI)X2
35	pRS426-2xHA Mga2-1-626; L31E, L32E, L35E, L36E
36	pRS426-2xHA Mga2-1-626; K254R
37	pRS426-2xHA Mga2-1-626; K254R; Δ2-130
38	pRS426-2xHA Mga2-1-626, K254R; Δ335-526

39	pRS426-2xHA Mga2-1-626; K254R; (LALKLAGLDI)X2
40	pRS426-2xHA Mga2-1-626; K254R; L31E, L32E, L35E, L36E
41	pRS426-2xHA Mga2-1-626; R252A, R253A, K254A
42	pRS426-Flag Mga2-1-626
43	pRS426-Flag Mga2-1-626; K254R
44	pRS426-Flag Mga2-1-626; R252A, R253A, K254A
45	pRS426-Flag Mga2-1-675
46	pRS426-GFP Mga2-1-626
47	pRS426-GFP Mga2-1-626; K254R
48	pRS426-GFP Mga2-1-626; R252A, R253A, K254A
49	pRS426-GFP-Mga2-1-626; R252A, R253A, K254A; Δ2-130
50	pRS426-GFP-Mga2-1-626; R252A, R253A, K254A; Δ335-526
51	pRS426-GFP-Mga2
52	pRS426-GFP-Mga2; R252A, R253A, K254A
53	Pk27-HIS-Sumo-Rsp5
54	Pk27-HIS-Sumo-Ubc4

Table S3: strains used in the study

Strain	genotype/description
BY4741	MATa; his3-Δ1 leu2Δ0 met15-Δ0 ura3-Δ0
mga2 KO	BY4741; Δmga2:G418
spt23 KO	BY4741; Δspt23:G418
2xHA-Mga2	BY4741
2xHA-Spt23	BY4741
2xHA-Mga2 (ΔAnk)	BY4741
pdr5 KO	BY4741; Δpdr5:G418
mga2spt23 double deletion	MATα; his3-Δ1 leu2Δ0 lys2Δ0 ura3-Δ0; Δspt23:kanMX4: Δmga2:natMX
4KO-LD; *mga2* KO	MATα; his3-Δ1 leu2Δ0 lys2Δ0 ura3-Δ0; are1::KanMX are2::KanMX lro1::loxP dga1::loxP; mga2Δ:hgh
NAB2-mCherry	MATa; his3-Δ1 leu2Δ0 met15-Δ0 ura3-Δ0; trp1Δ:natMX; NAB2-mCherry:TRP1
